# Antimicrobial activities of stearidonic and gamma-linolenic acids from the green seaweed *Enteromorpha linza* against several oral pathogenic bacteria

**DOI:** 10.1186/1999-3110-54-39

**Published:** 2013-09-25

**Authors:** Nam-Hee Park, Jae-Suk Choi, Seon-Yeong Hwang, Yang-Chun Kim, Yong-Ki Hong, Kwang Keun Cho, In Soon Choi

**Affiliations:** 1 Gijang Local Products Co. Ltd, Ilgwang-myeon, Gijang-gun, Busan, 619-911 Republic of Korea; 2 RIS Center, IACF, Silla University, Sasang-gu, Busan, 617-736 Republic of Korea; 3 Department of Biotechnology, Pukyong National University, Nam-gu, Busan, 608-737 Republic of Korea; 4 Department of Animal Resources Technology, Gyeongnam National University of Science and Technology, Jinju, Gyeongnam, 660-758 Republic of Korea; 5 Depertment of Biological Science, Silla University, Sasang-gu, Busan, 617-736 Republic of Korea

**Keywords:** Stearidonic acid, Gamma-linolenic acid, Antimicrobial activity, *Enteromorpha linza*, *Prevotella intermedia*, *Porphyromonas gingivalis*, Oral pathogen

## Abstract

**Background:**

We found that the edible green seaweed *Enteromorpha linza* displayed potent antimicrobial activity against *Prevotella intermedia* and *Porphyromonas gingivalis*. To elucidate the active component of *E. linza*, isolation procedures were performed.

**Results:**

The main active compound was isolated by polarity fractionation, Sephadex LH-20 gel chromatography, and reverse-phase high-performance liquid chromatography (RP-HPLC). The active compounds were eluted at isocratic 95% acetonitrile by RP-HPLC and identified as unsaturated fatty acids, stearidonic acid (SA, C18:4 n-3) and gamma-linolenic acid (GLA, C18:3 n-6) by gas chromatography–mass spectrometry, ^1^H nuclear magnetic resonance (NMR) spectroscopy, and ^13^C NMR spectroscopy. The yields of SA and GLA from dried seaweed tissue were 6.33 × 10^-3^% and 6.47 × 10^-3^%, respectively. The minimal inhibitory concentration values of SA and GLA were 39.06 μg/mL against *P. intermedia* and 9.76 μg/mL against *P. gingivalis*, respectively. SA and GLA were also active against several other oral pathogens, including *Aggregatibacter actinomycetemcomitans, Candida albicans, Fusobacterium nucleatum* subsp. *vincenti*, and *Streptococcus mutans*, at micromolar concentrations.

**Conclusions:**

These data suggest that the *E. linza* extracts SA and GLA are useful antimicrobial agents for the prevention and/or treatment of periodontitis.

**Electronic supplementary material:**

The online version of this article (doi:10.1186/1999-3110-54-39) contains supplementary material, which is available to authorized users.

## Background

Periodontitis is a chronic inflammatory disease initiated by a group of gram-negative periodontal pathogens, including *Prevotella intermedia* and *Porphyromonas gingivalis.* Systemic or topical antibiotic therapies have been found to be useful in the treatment of periodontal disease (Slots and Ting 2002). However, antibiotics are commonly known to induce side effects (Falagas and Siakavellas 2000) and the development of bacterial resistance (Lopez and Gerwick 1988). Thus, for many years, numerous attempts have been made to develop new agents capable of preventing and/or treating periodontitis (Park et al. 2005).

Interest in marine organisms as natural sources of pharmaceutical agents has increased recently (Newman et al. 2003). Seaweeds produce many secondary metabolites with a variety of activities; compounds with antiviral, antifungal, and antibacterial activity have been detected from various marine algae (del Val et al. 2001; Newman et al. 2003).

In our previous work, we found that the edible green seaweed *Enteromorpha linza* displays potent antimicrobial activity against *P. intermedia* and *P. gingivalis* without side effects at a moderate dose (Choi et al. 2012). Additionally, a mouth rinse containing *E. linza* extract has shown clinical effects against gingivitis, as measured by the plaque index (PI), gingival index (GI), bleeding on probing (BOP), and activity against two bacterial strains (*P. intermedia* and *P. gingivalis*) (Cho et al. 2011). This mouth rinse produced effects similar to those of Listerine®. To discover therapeutic agents against periodontitis from the seaweed with few or no side effects and potent antimicrobial activity, we isolated and identified active antimicrobial compounds from *E. linza* extract and present data regarding its antimicrobial activity against several oral pathogens.

## Methods

### Algae

The green seaweed *E. linza* was collected from the seashore near Ildo-dong (33°31′15.06″N, 126°32′54.70″E), Cheju Island, Korea, in April 2009. After rinsing with tap water to remove salt and miscellanea, the samples were dried for 1 day at room temperature using an electric fan. Samples were then ground to a powder using a coffee grinder for 5 min and stored at −20°C until use.

### Reagents

High-performance liquid chromatography (HPLC)-grade reagents (or the highest grade available) and culture media were purchased from Sigma Aldrich, Ltd. (Poole, Dorset, UK). All solutions were made with ultrapure deionized water (Milli-Q advantage A10 ultrapure water purification system; Millipore, Billerica, MA, USA).

### Isolation of antimicrobial compounds

Approximately 150 g of dried *E. linza* powder was extracted with a 600-mL solution of methanol–water (4:1) at room temperature overnight. The extract was then filtered through Toyo No. 2 filter paper under reduced pressure. This extraction procedure was repeated three times and the extracts were combined. The methanol–water extract was concentrated to a dark-green residue (30.2 g) under reduced pressure in an evaporator. The extract was fractionated according to polarity, as described by Harborne (1998). The fraction was acidified to pH 3.0 using 2 M sulfuric acid and extracted three times with chloroform, resulting in a moderately polar extract that contained the main antimicrobial activity. This fraction III (4.4 g) was loaded on a Sephadex LH-20 (Sigma LH20100; Sigma Aldrich, Ltd.) gel column (2.2 × 100 cm) using 100% MeOH as the eluent. Each 10-mL fraction was collected at a flow rate of 1 mL/min. The active fraction, designated as S14 (173 mg), was dried and dissolved in 17.3 mL MeOH for reverse-phase high-performance liquid chromatography (RP-HPLC). Separation of the three major peaks (S14–1, S14–2, and S14-3) was achieved using an Alltima C18 column (10 mm ID × 25 cm) (W. R. Grace & Co., Columbia, MD, USA). Analysis was performed on a Waters 600 gradient liquid chromatograph and a Waters 2707 autosampler (Waters Co., Milford, MA, USA) monitored at 197 nm. The mobile phase consisted of two solvent systems: acetonitrile with 0.1% trifluoroacetic acid (TFA) and distilled water with 0.1% TFA. Elution was performed with an isocratic of 95% acetonitrile with 0.1% TFA (5% distilled water added) for 15 min at a flow rate of 4 mL/min. Purification was performed using the same system to yield the pure compound (S14-1: 8.8 mg; S14–2: 9.5 mg; S14–3: 9.7 mg). Each eluted compound was dried under a stream of nitrogen gas.

### Analytical methods

The purified compound was analyzed on a nuclear magnetic resonance (NMR) spectrometer (JNM-ECP 400; JEOL, Tokyo, Japan), operating at 500 and 100 MHz for ^1^H and ^13^C, respectively, using methanol-d (CD_3_OD). Gas chromatography–mass spectrometry (GC–MS) was performed on a 5975C gas chromatograph (Agillent, Santa Clara, CA, USA) equipped with an HP-5MS capillary column (30 m × 250 μm × 0.25 μm). The column temperature was programmed from an initial temperature of 80°C for 2 min and raised to a final temperature of 300°C at a rate of 5°C/min. The samples were converted to methyl ester derivatives by the protocol reported by Lepage and Roy (1986). The injection volume was 1 μL, the carrier gas was helium (flow rate, 1 mL/min) and the split ratio was 20:1. A computerized search was performed to identify a standard sample for comparison (Sigma 56463: methyl stearidonate solution; Sigma L6503: methyl γ-linolenate solution for GC) (Sukatar et al. 2006). The structure of the purified compound was inferred by comparison with C_18_H_28_O_2_ and C_18_H_30_O_2_ structures from a NMR spectra database (Aires-de-Sousa et al. 2002).

### Test microorganisms and culture media

*In vitro* antimicrobial activity assays were performed with the anaerobic bacteria of *P. intermedia* KCTC25611 and *P. gingivalis* KCTC381 obtained from the Korean Collection for Type Culture (Deajeon, Korea). These strains were cultivated on tryptic soy agar (Difco, Sparks, MD, USA) media containing 10% sheep blood, 0.1% hemin (Sigma, St. Louis, MO, USA) and vitamin K1 (Sigma, USA) in a Bactron IV chamber (SHELLAB, Cornelius, OR, USA) at 37°C, 5% CO_2_, 10% H_2_, and 85% N_2_.

### Antimicrobial activity against oral pathogens

Following identification of the active compounds, their antimicrobial activity was measured against several oral pathogens, including anaerobic *Aggregatibacter actinomycetemcomitans* KCTC 3698, *Candida albicans* KCTC 17485*, Fusobacterium nucleatum* subsp. *vincenti* KCTC 5105, and *Streptococcus mutans* KCTC 3065. *A. actinomycetemcomitans* was cultivated on brucella medium (Sigma, USA) containing 3% horse serum (Sigma, USA) in a Bactron IV chamber. *C. albicans* was cultivated on RPMI 1640 medium (Sigma, USA), *F. nucleatum* subsp. *vincenti* was cultivated on Schaedler medium (Sigma, USA), and *S. mutans* was cultivated on BHI medium (Sigma, USA) with 3% horse serum (Sigma, USA). All microorganism incubations were performed at 37°C.

### Determination of minimal inhibitory concentration (MIC) values

The Determination of MIC values were performed as described previously (Choi et al. 2012). Inocula were prepared from 24-h broth cultures and then diluted to ten volume of the original volume and adjusted to the 0.5 McFarland standard solution turbidity containing approximately 1 to 2 × 10^8^ CFU/mL. Microtitre plates were prepared by dispensing the inoculum and active compound into each well. The first well of each strip contained 100 μL broth with no compound and 100 μL inoculum, and represented the negative control. The second well on each strip contained 90 μL broth, 10 μL methanol, and 100 μL inoculum, and represented the vechicle control. The final volume in each well was 200 μL. Following incubation, the MIC value was defined as the lowest concentration that yielded no bacterial cell growth (National Committee for Clinical Laboratory Standards 2004). All MIC tests were performed independently in triplicate. The antimicrobial agent’s triclosan-methyl (Sigma-Aldrich 34228) and chlorhexidine (Sigma-Aldrich 282227) were included in the assays as positive controls.

### Quantification of the compound

To measure the levels of antimicrobial compounds in *E. linza*, the thalli were completely dried in the shade at room temperature for 1 week and ground into a powder for 5 min. The powder (0.5 g) was extracted with 10 mL dichloromethane on a rotator for 1 h at 30 rpm. Following centrifugation at 2,000 × *g* for 5 min, 4 mL of the clean supernatant was evaporated to 5 mg/mL for RP-HPLC. Each 100-μL aliquot was separated on an Alltima C18 column (10 mm ID × 25 cm) (W. R. Grace & Co.) using the same isolation procedure. The compound levels were assessed by measuring the dimensions of the HPLC peaks using the standard curve of the pure compound.

## Results

### Isolation of antibacterial fractions

**Figure 1:**
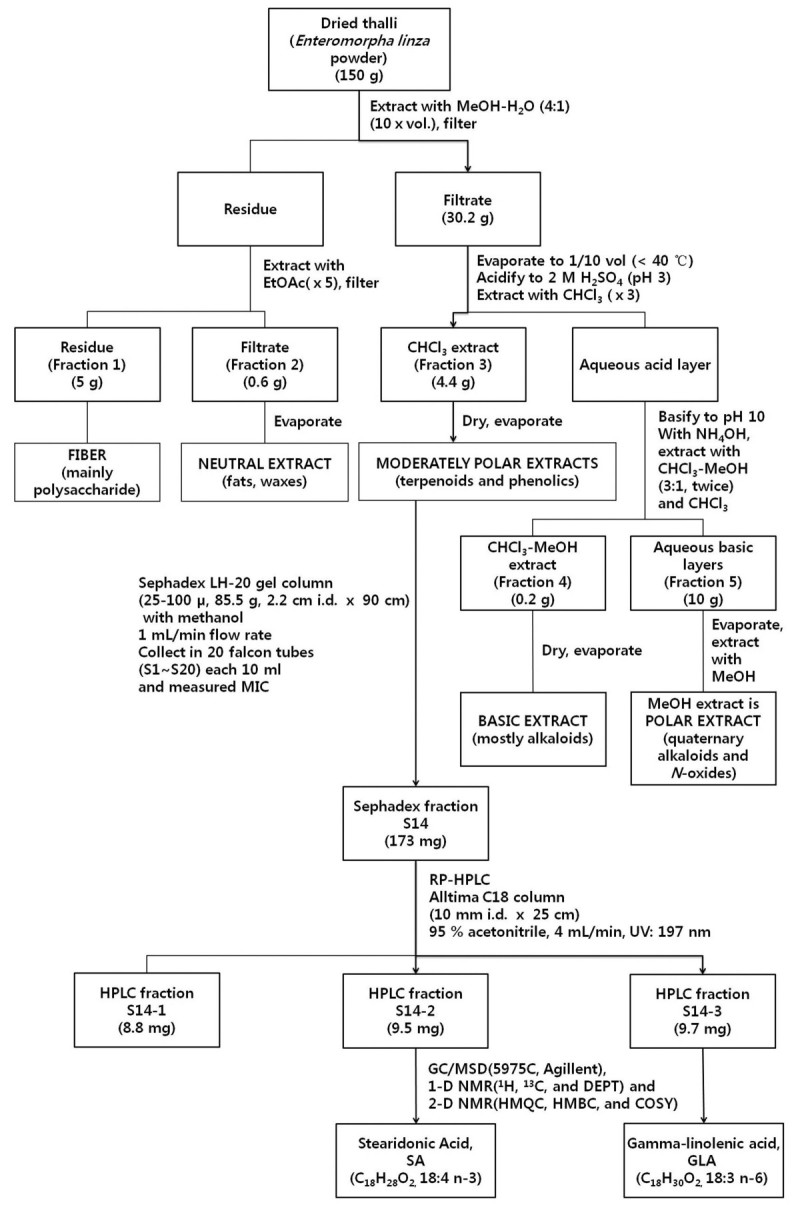
**Purification of antimicrobial compounds isolated from** ***Enteromorpha linza*** **.** ↓: purification of stearidonic and gamma-linolenic acids.

The purification procedure is shown in Figure 1. The active compounds were obtained through methanol extraction, chloroform fractionation, Sephadex fractionation (S14), and HPLC fractionation (S14-2 and S14-3). Fraction 14 from the Sephadex fractionation of the chloroform fraction was found to be antibacterial against both *P. intermedia* and *P. gingivalis*. Fraction S14 was further fractionated by RP-HPLC. The active compounds were eluted at 5.1 min (S14-1), 6.3 min (S14-2), and 7.9 min (S14-3), respectively, in isocratic 95% acetonitrile by RP-HPLC. Among them, the antimicrobial activities of S14-2 and S14-3 were much stronger than those of S14-1. Thus, further isolation was conducted using S14-2 and S14-3, which were oily and yellow. The weights of S14-2 and S14-3 were 9.5 mg and 9.7 mg, and the yields were 6.33 × 10^-3^% and 6.47 × 10^-3^%, respectively, from the dried seaweed powder. The antibacterial potency of the purified compounds was determined by measuring MIC values. The MIC values of the extracts and fractionates were measured against the anaerobic bacteria of *P. intermedia and P. gingivalis* (Table 1). The MIC values of isolated S14-2 [stearidonic acid (SA)] and S14-3 [gamma-linolenic acid (GLA)] showing the same MIC values were 39.06 μg/mL against *P. intermedia* and 9.76 μg/mL against *P. gingivalis*.

**Table 1:** **Minimum inhibitory concentration (MIC) values and purification factor of the extract and fraction type against** ***P. intermedia*** **and** ***P. gingivalis***

Extract and fraction type	***P. intermedia***		***P. gingivalis***		Yield (%)
	MIC (μg/mL)	Purification factor	MIC (μg/mL)	Purification factor	
*E. linza* powder	-	-	-	-	100
Methanol extract	625^*^	1	312.50^*^	1	20.10
Fraction III (chloroform fraction)	625	2^1^	156.25	2^3^	2.93
Sephadex fraction No. S14	78.12	2^4^	19.53	2^6^	0.12
HPLC fraction No. S14-1	156.25	2^3^	39.06	2^5^	0.0058
HPLC fraction No. S14-2 (SA)	39.06	2^5^	9.76	2^7^	0.0063
HPLC fraction No. S14-3 (GLA)	39.06	2^5^	9.76	2^7^	0.0064

*HPLC* high-performance liquid chromatography, *SA* stearidonic acid, *GLA* gamma-linolenic acid.

* The MIC values of methanol extract were adapted from Choi et al. (2012).

-: Not determined.

### Identification of antibacterial compounds

**Figure 2:**
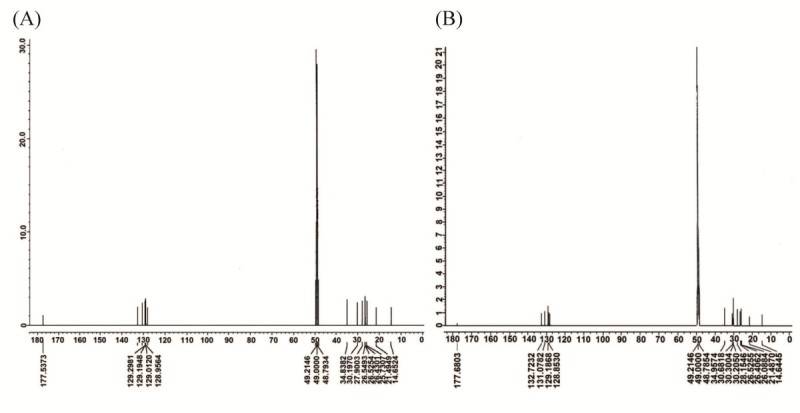
^**13**^**C nuclear magnetic resonance spectra of the antimicrobial fractions.** ^13^C nuclear magnetic resonance spectra obtained in methanol-d (CD_3_OD) at 500 MHz from the antimicrobial fractions **(A)** S14-2 (stearidonic acid) and **(B)** S14-3 (gamma-linolenic acid) from reverse-phase, high-performance liquid chromatographic separation.

**Figure 3:**
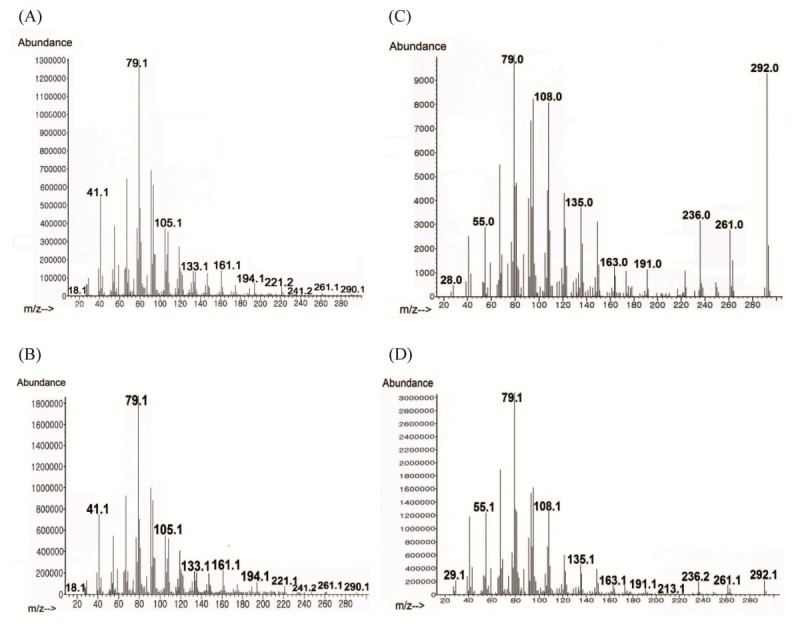
**Gas chromatography–mass spectrometry spectra of antimicrobial fractions. (A)** stearidonic acid, methyl ester (standard), **(B)** S14-2, methyl ester **(C)** linolenic acid, methyl ester (standard), and **(D)** S14-3, methyl ester from reverse-phase, high-performance liquid chromatographic separations that completely superimposed over those of commercial compounds.

**Figure 4:**
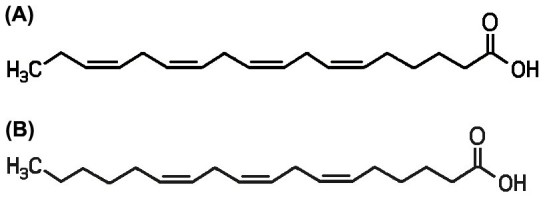
**Structures of purified compounds. (A)** stearidonic acid (C18:4 n-3) and **(B)** gamma-linolenic acid (C18:3 n-6).

Assignments were performed by analyzing heteronuclear multiple-quantum correlation (HMQC), heteronuclear multiple-bond correlation (HMBC), and correlation spectroscopy (COSY) spectra. Infrared analysis of the purified compounds showed absorption for OH and carbonyl function. The ^1^H-NMR data (Table 2) and ^13^C-NMR spectra (Figure 2) of S14-2 (SA) and S14-3 (GLA) were characteristic of an unsaturated fatty acid in its free form. The GC–MS spectra (Figure 3) of S14-2 (methyl ester) and S14-3 (methyl ester) were completely superimposed over those of the commercial SA (methyl ester) and γ-linolenic acid (methyl ester). From these spectral data, we identified the compound as the polyunsaturated fatty acids (PUFAs) 6,9,12,15-octadecatetraenoic acid or SA (C18:4 n-3; Figure 4A) and 6,9,12-octadecatrienoic acid or GLA (C18:3 n-6; Figure 4B).

**Table 2:** ^**1**^ **H nuclear magnetic resonance (NMR) data of the antimicrobial fractions:** ^**1**^ **H NMR data obtained in methanol-d (CD**_**3**_ **OD) at 500 MHz from the antimicrobial fractions (a) S14–2 (stearidonic acid) and (b) S14–3 (gamma-linolenic acid) from reverse-phase, high-performance liquid chromatographic separation**

(a)			(b)		
	^1^H	Peak label on ^1^H-NMR spectrum		^1^H	Peak label on ^1^H-NMR spectrum
C1	-	-	C1	-	-
C2	2.27, t, 2H	e	C2	2.24–2.26, t, 2H	e
C3	1.61, m, 2H	c	C3	1.57–1.60, m, 2H	c
C4	1.41, m, 2H	b	C4	1.32, m, 2H	b
C5	2.08–2.10, m, 2H	d	C5	2.04–2.09, m, 2H	d
C6	5.34–5.36, m, 1H	g	C6	5.32–5.34, m, 1H	g
C7	5.34–5.36, m, 1H	g	C7	5.32–5.34, m, 1H	g
C8	2.81–2.83, m, 2H	f	C8	2.78–2.84, m, 2H	f
C9	5.34–5.36, m, 1H	g	C9	5.32–5.34, m, 1H	g
C10	5.34–5.36, m, 1H	g	C10	5.32–5.34, m, 1H	g
C11	2.81–2.83, m, 2H	f	C11	2.78–2.84, m, 2H	f
C12	5.34–5.36, m, 1H	g	C12	5.32–5.34, m, 1H	g
C13	5.34–5.36, m, 1H	g	C13	5.32–5.34, m, 1H	g
C14	2.81–2.83, m, 2H	f	C14	2.04–2.09, m, 2H	d
C15	5.34–5.36, m, 1H	g	C15	1.32, m, 2H	b
C16	5.34–5.36, m, 1H	g	C16	1.32, m, 2H	b
C17	2.08–2.10, m, 2H	d	C17	1.32, m, 2H	b
C18	0.97, t, 3H	a	C18	0.96, t, 3H	a

### Antimicrobial activity of SA and GLA against several oral pathogens

When the antimicrobial activities of the purified SA (S14–2) and GLA (S14-3) and the commercially available SA (Sigma-Aldrich 49509) and GLA (Sigma-Aldrich L2378) against *P. intermedia* and *P. gingivalis* were compared, we observed no difference. The commercially available SA and GLA were then tested for antimicrobial activity against several oral pathogens, including *A. actinomycetemcomitans, C. albicans, F. nucleatum* subsp. *vincenti*, and *S. mutans* (Table 3). The MIC values of SA were 312.50 μg/mL against *A. actinomycetemcomitans*, 312.50 μg/mL against *C. albicans*, 39.06 μg/mL against *F. nucleatum* subsp. *vincenti*, and 1,250 μg/mL against *S. mutans*. The MIC values of GLA were 78.12 μg/mL against *A. actinomycetemcomitans*, 78.12 μg/mL against *C. albicans*, 9.76 μg/mL against *F. nucleatum* subsp. *vincenti,* and 625 μg/mL against *S. mutans*. Triclosan and chlorhexidine, which are broad-spectrum antimicrobial agents found in toothpaste, soap, deodorant, detergents, cosmetics, pharmaceuticals, plastics, and fabrics (Nudera et al. 2007; Haraszthy et al. 2006), were used as positive controls. The MIC values of triclosan were not determined against *A. actinomycetemcomitans*, 312.50 μg/mL against *C. albicans*, 4.88 μg/mL against *F. nucleatum* subsp. *vincenti*, not determined against *S. mutans*, 1,250 μg/mL against *P. intermedia*, and 78.12 μg/mL against *P. gingivalis*. The MIC values of chlorhexidine were 4.88 μg/mL against *A. actinomycetemcomitans*, 4.88 μg/mL against *C. albicans*, 2.44 μg/mL against *F. nucleatum* subsp. *vincenti*, 9.76 μg/mL against *S. mutans*, 1.22 μg/mL against *P. intermedia*, and 1.22 μg/mL against *P. gingivalis*. The MIC of triclosan was 23 μg/mL for *P. intermedia* and 6 μg/mL for *P. gingivalis*, and the MICs of chlorhexidine were 67.5 μg/mL and 125 μg/mL, respectively. Extracts and volatile oil of *E. linza* showed no activity against the yeast *C. albicans* (Sukatar et al. 2006). Similarly, the methanol extracts of *E. compressa*, *E. muscoides*, and *Ulva rigida* displayed no activity against *C. albicans* (del Val et al. 2001).

**Table 3:** **Minimum inhibitory concentration (MIC) values of commercial stearidonic acid (SA) and gamma-linolenic acid (GLA), and positive controls (triclosan and chlorhexidine) against several oral pathogens**

Oral pathogens	MIC value (μg/mL)			
	Positive controls		SA	GLA
	Triclosan	Chlorhexidine		
*Aggregatibacter actinomycetemcomitans*	ND	4.88	312.50	78.12
*Candida albicans*	312.50	4.88	312.50	78.12
*Fusobacterium nucleatum* subsp. *vincenti*	4.88	2.44	39.06	9.76
*Streptococcus mutans*	ND	9.76	1,250	625
*Prevotella intermedia*	1,250	1.22	39.06	39.06
*Porphyromonas gingivalis*	78.12	1.22	9.76	9.76

All measurements were performed in triplicate, and are the average of three experiments; *ND* not determined.

### Quantification of the compound

To quantify the active compounds directly from the seaweed tissue, 0.0131 g (2.62%) of dichloromethane extract was obtained from 0.5 g of powder. The S14–2 and S14–3 peaks were integrated using the Empower™ software (Waters Co.), and the percentile dimensions were measured as 18.87% and 5.22% by HPLC. The amounts of S14–2 and S14–3 were calculated as 2.5 mg (0.50%) and 0.7 mg (0.14%) from 0.5 g of *E. linza* powder.

## Discussion

Periodontitis is a common chronic inflammatory disease caused by the accumulation of a bacterial matrix at the gum line (Socransky et al. 1999). It is characterized by gum tissue separation from the tooth, which forms a periodontal pocket that leads to bone and tooth loss. Traditional antibiotic therapies for periodontitis target the bacterial infection, which may be the initiating event responsible for the ensuing inflammation and tissue destruction. However, antibiotics are known to induce side effects (Falagas and Siakavellas 2000) and lead to the development of bacterial resistance (Lopez and Gerwick 1988). In this study, we attempted to identify a new therapeutic agent with few or no side effects and potent antimicrobial activity from a seaweed source. Seaweed is abundant and produces diverse biologically active substances. In our previous screening, we found that green seaweed (*E. linza*) extract displayed potent antimicrobial activity against *P. intermedia* and *P. gingivalis* (Choi et al. 2012). In this study, the active compounds were identified as the unsaturated fatty acids SA (C18:4 n-3) and GLA (C18:3 n-6) by GC–MS, ^1^H NMR, and ^13^C NMR spectroscopy. SA and GLA were found to be active against several oral pathogens.

Fatty acids of linolenic acid (Ohta et al. 1994), palmitoleic acid and hexadecatrienoic acid (HTA) (Desbois et al. 2008), and eicosapentaenoic acid (EPA) (Desbois et al. 2009) were also identified as antimicrobial agents against several human pathogens. SA isolated from *Undaria pinnatifida* has been found to be active against *S. mutans* (Yun et al. 2007). Several commercial the ω-3 and ω-6 polyunsaturated fatty acids (PUFAs) were also identified as antimicrobial agents against stomatitis, periodontitis and gingivitis (Choi et al. 2013). Thus, free fatty acids may hold promise as a new topical or systemic treatment for periodontitis.

PUFA toxicity has been attributed to its detergent activity, which disrupts bacterial membranes and the formation of short-chain aldehydic compounds by autooxidation (Knapp and Melly 1986), and selectively inhibits bacterial enoyl-acyl carrier reductase, an essential component of bacterial fatty acid synthesis (Zheng et al. 2005). As a highly unsaturated (n-3) PUFA, the unsaturation index of SA is lower than those of EPA and docosahexaenoic acid (DHA); SA thus potentially displays improved stability characteristics, enhancing its commercial value (Guil-Guerrero 2007; Whelan 2009). GLA is produced in the body as an intermediate in the metabolism of linoleic acid (LA), an essential fatty acid of the omega-6 series, by the action of delta-6-desaturase. GLA has gained importance due to its anti-inflammatory and anti-cancer action, and has been shown to improve nerve conduction velocity in patients with diabetes, leading to improved blood flow and reduced tingling of the extremities (Kapoor and Huang 2006). The topical application of bioactive products derived from n-3 fatty acids has conferred dramatic protection against inflammation-induced tissue and bone loss associated with periodontitis in experimental models (Naqvi et al. 2010). Indeed, GLA displayed a protective effect against periodontitis due to its anti-inflammatory properties in randomized controlled human trials (Rosenstein et al. 2003). In the case of SA, despite its anti-inflammatory activity (Khan et al. 2007), its protective effect against periodontitis has not been confirmed.

The safety of SA was investigated in human studies of SA-containing oils. When an SA intake ranging from 1 to 4 g/d was investigated in 28-d and 90-d rats, doses of up to 4 g/kg body weight daily produced no SA-specific adverse effect. Furthermore, SA-enriched soybean oil has recently been obtained and is generally recognized as safe (GRAS) for use as a food ingredient in the United States (Lemke et al. 2010). As GLA is present in high levels in maternal breast milk (Puri 2007), it would be expected to be a safe product when used in moderation.

## Conclusions

In conclusion, we have shown for the first time that the fatty acids SA and GLA inhibit the growth of oral pathogens. Moreover, mouth rinses containing the *E. linza* extract are effective against gingivitis disease (Cho et al. 2011), and the fatty acids of SA (Khan et al. 2007) and GLA (Kapoor and Huang 2006) display anti-inflammatory properties. To date, no bacterial resistance to free fatty acids has been encountered and no resistance phenotype has emerged (Desbois et al. 2009). Thus, this study presents a strategy for the prevention and/or treatment of oral pathogen infection with minimal side effects and no bacterial resistance.

## Authors’ original submitted files for images

Below are the links to the authors’ original submitted files for images. Authors’ original file for figure 1 Authors’ original file for figure 2 Authors’ original file for figure 3 Authors’ original file for figure 4
