# Supplementary figures and images for: Antimicrobial activities of stearidonic and gamma-linolenic acids from the green seaweed Enteromorpha linza against several oral pathogenic bacteria
Source: Bot Stud. 2013 Sep 25;54:39. doi: 10.1186/1999-3110-54-39 (PMC5432978; doi:10.1186/1999-3110-54-39)

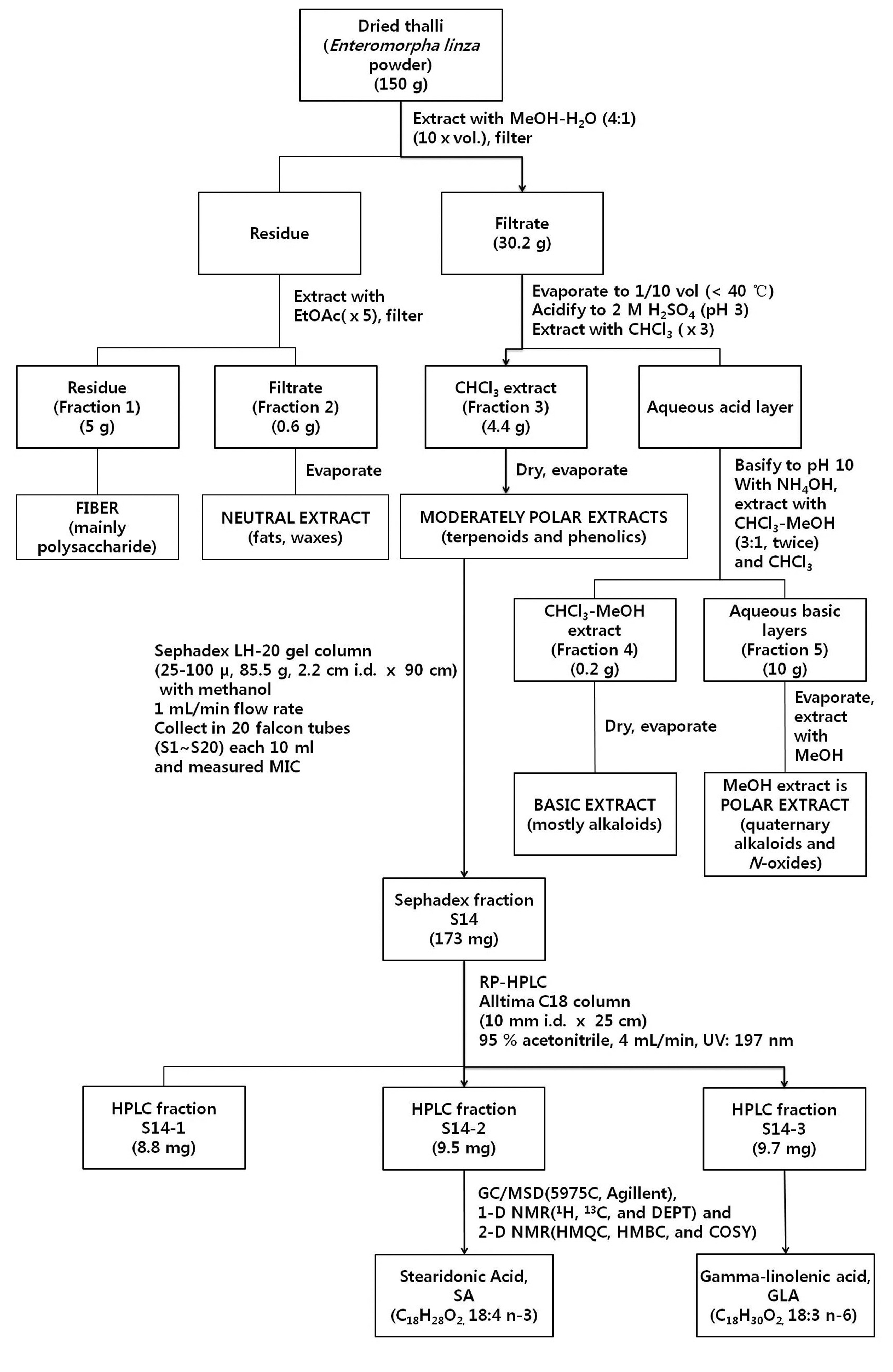

Supplement: Supplementary file 1 — Authors’ original file for figure 1 [file 40529_2013_35_MOESM1_ESM.jpeg]

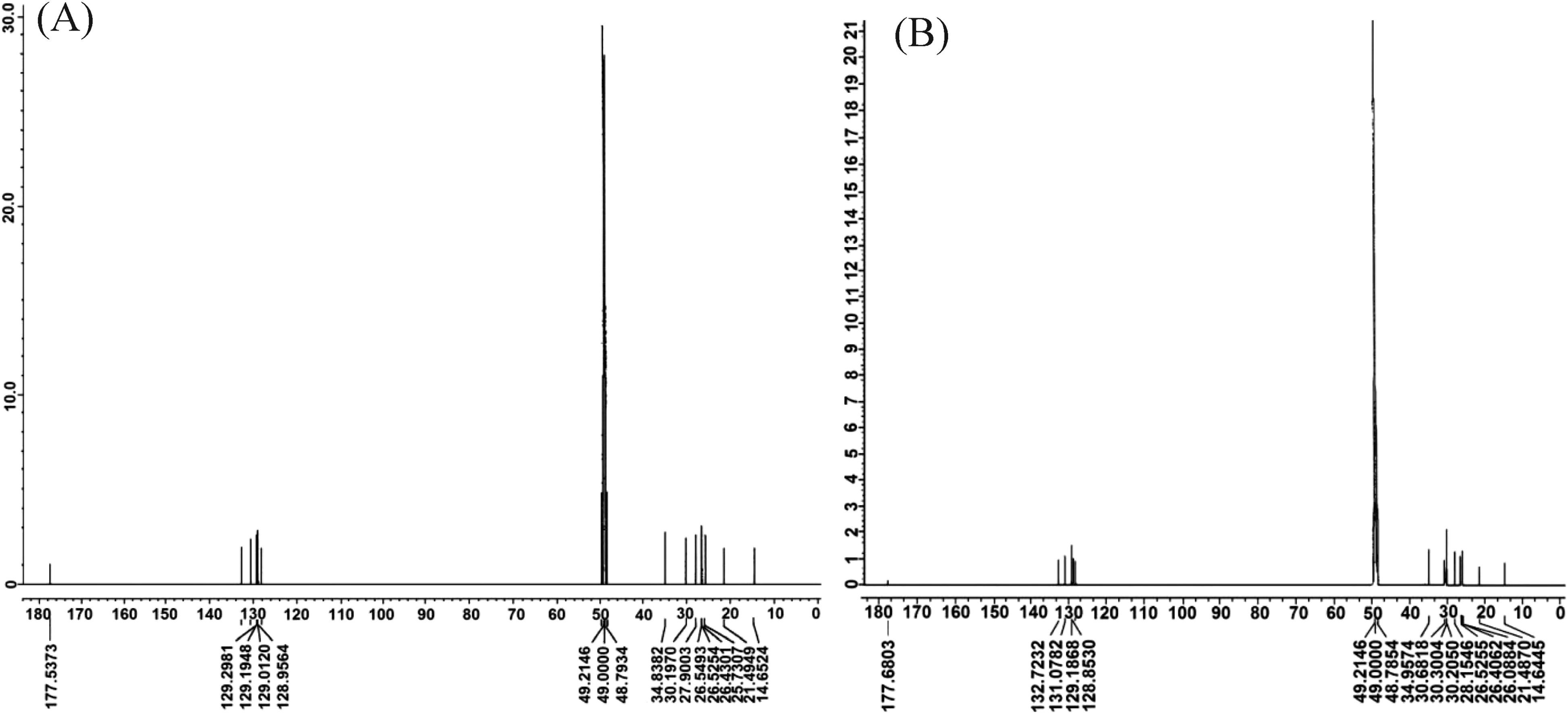

Supplement: Supplementary file 2 — Authors’ original file for figure 2 [file 40529_2013_35_MOESM2_ESM.tiff]

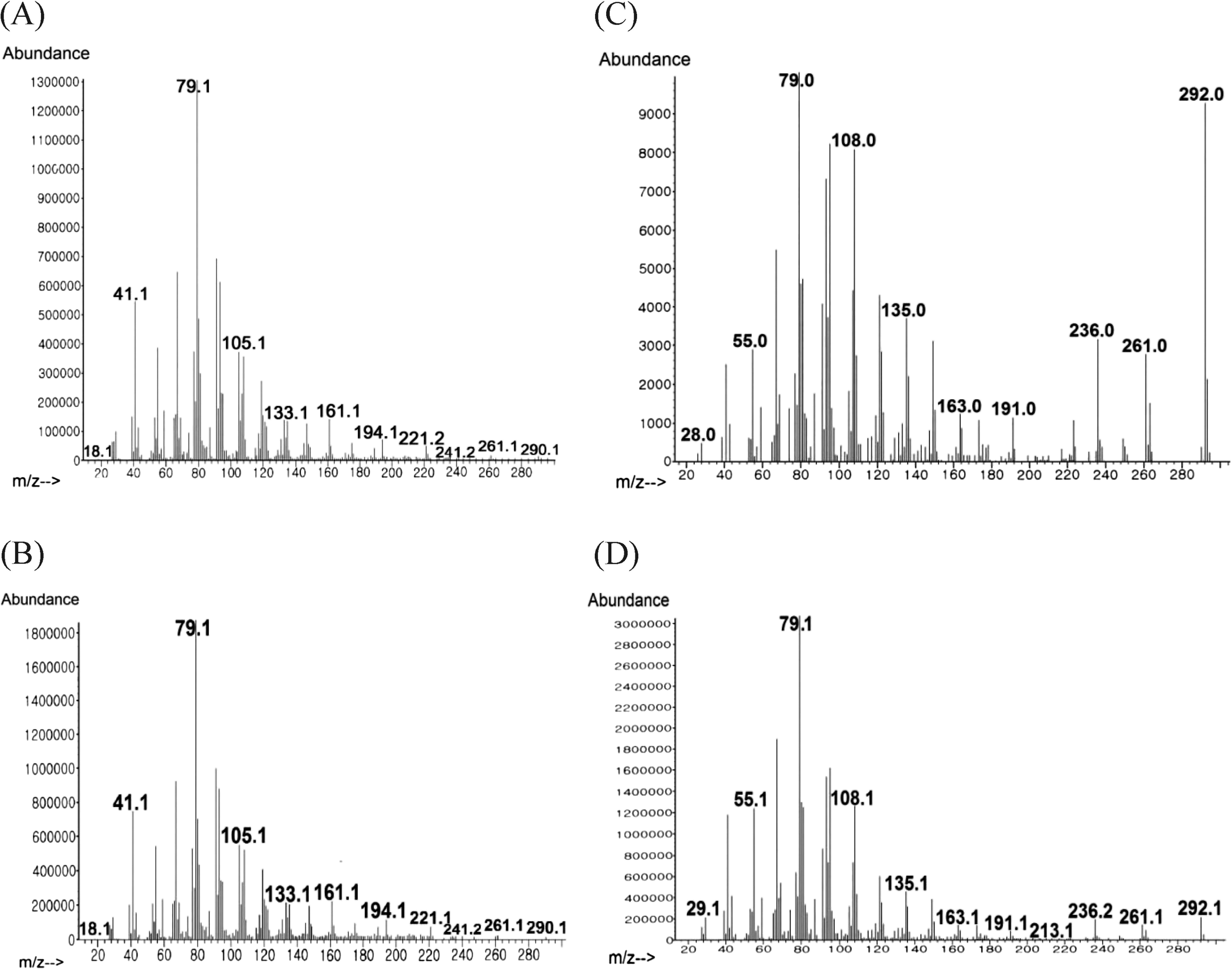

Supplement: Supplementary file 3 — Authors’ original file for figure 3 [file 40529_2013_35_MOESM3_ESM.tiff]

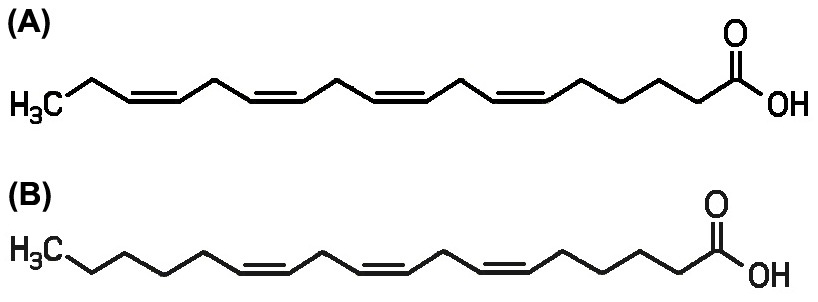

Supplement: Supplementary file 4 — Authors’ original file for figure 4 [file 40529_2013_35_MOESM4_ESM.jpeg]
